# The Multifaceted Functions of CXCL10 in Cardiovascular Disease

**DOI:** 10.1155/2014/893106

**Published:** 2014-04-23

**Authors:** Pleunie van den Borne, Paul H. A. Quax, Imo E. Hoefer, Gerard Pasterkamp

**Affiliations:** ^1^Laboratory of Experimental Cardiology, University Medical Center Utrecht, P.O. Box 85500, 3508 GA, Utrecht, The Netherlands; ^2^Department of Surgery, Leiden University Medical Center, P.O. Box 9600, 2300 RC, Leiden, The Netherlands; ^3^Einthoven Laboratory of Experimental Vascular Medicine, Leiden University Medical Center, P.O. Box 9600, 2300 RC, Leiden, The Netherlands; ^4^Interuniversity Cardiology Institute of the Netherlands, P.O. Box 19258, 3501 DG, Utrecht, The Netherlands

## Abstract

C-X-C motif ligand 10 (CXCL10), or interferon-inducible protein-10, is a small chemokine belonging to the CXC chemokine family. Its members are responsible for leukocyte trafficking and act on tissue cells, like endothelial and vascular smooth muscle cells. CXCL10 is secreted by leukocytes and tissue cells and functions as a chemoattractant, mainly for lymphocytes. After binding to its receptor CXCR3, CXCL10 evokes a range of inflammatory responses: key features in cardiovascular disease (CVD). The role of CXCL10 in CVD has been extensively described, for example for atherosclerosis, aneurysm formation, and myocardial infarction. However, there seems to be a discrepancy between experimental and clinical settings. This discrepancy occurs from differences in biological actions between species (e.g. mice and human), which is dependent on CXCL10 signaling via different CXCR3 isoforms or CXCR3-independent signaling. This makes translation from experimental to clinical settings challenging. Furthermore, the overall consensus on the actions of CXCL10 in specific CVD models is not yet reached. The purpose of this review is to describe the functions of CXCL10 in different CVDs in both experimental and clinical settings and to highlight and discuss the possible discrepancies and translational difficulties. Furthermore, CXCL10 as a possible biomarker in CVD will be discussed.

## 1. Introduction

Chemokines are soluble low molecular weight proteins that are involved in a wide variety of processes during physiological and pathological conditions. They can be secreted by and act on different cell types depending on the expression of specific receptors. Chemokines are known to be involved in leukocyte trafficking but can also act on other cells like endothelial cells and vascular smooth muscle cells (VSMCs) [1]. Subgroups of chemokines that have been identified are C, CC, CX3C, and CXC, based on molecular structure and arrangement of cysteine residues that form disulfide-bonding pairs. C chemokines mainly recruit lymphocytes, while CC chemokines recruit monocytes. So far, only one CX3C chemokine has been described. CX3CL1 (fractalkine) can act as a chemoattractant for different leukocytes (soluble CX3CL1) and promotes cell adhesion to activated endothelial cells (cell-bound CX3CL1). The last family of chemokines, the CXC chemokines, is involved in leukocyte trafficking and endothelial and vascular smooth muscle cell (VSMC) proliferation and motility [2–4]. In this review, the role of C-X-C motif ligand 10 (CXCL10) in different cardiovascular disease models will be highlighted. CXCL10 belongs to the CXC chemokine family [4]. The CXC chemokines can be subdivided into two groups according to the presence or absence of a tripeptide glutamic acid-leucine-arginine (Glu-Leu-Arg motif; “ELR”) motif preceding the first conserved cysteine: the ELR motif positive (ELR+) and ELR motif negative (ELR−) CXC chemokines. ELR+ CXC chemokines are known to attract neutrophils and hold more angiogenic properties, whereas ELR− CXC chemokines are lymphocyte attractants with angiostatic properties [5, 6]. CXCL10 belongs to the ELR− CXC chemokines and is also known as interferon-inducible protein-10 (IP-10). As the name implies, this chemokine can be secreted upon interferon gamma (IFN*γ*) production by a wide variety of cell types, such as endothelial cells, fibroblasts, keratinocytes, monocytes, and T lymphocytes [7], but secretion can also be induced by lipopolysaccharide and proinflammatory cytokines such as IFN-alpha and IFN-beta as well as tumor necrosis factor-alpha [8, 9], depending on the cell type. CXCL10 exerts its biological effects mainly via binding to CXCR3 [10]. Also, CXCR3-independent pathways have been studied for CXCL10 involving binding of CXCL10 to heparan sulfate glycosaminoglycans (GAGs) [11–14] for cells not expressing CXCR3. CXCL10 not only induces chemoattraction of inflammatory cells, but also migration and proliferation of endothelial cells and VSMCs [15–18]. CXCL10 has been studied extensively in cardiovascular diseases, both experimentally and clinically.

The aim of this review is to summarize the role of CXCL10 in cardiovascular disease in both experimental and clinical studies and highlight the discrepancies between the different settings.

## 2. CXCL10 Signaling and Effects

### 2.1. CXCL10 Signaling

CXCL10 signals via binding to its receptor CXCR3. Next to CXCL10, CXCL9 and CXCL11 can also bind to this receptor. CXCR3 is a seven* trans*-membrane-spanning G-protein-coupled receptor (GPCR). This receptor is composed of the G*α*, B*β*, and G*γ* subunit. Binding of a ligand to CXCR3 leads to the exchange of guanosine triphosphate (GTP) to guanosine phosphate (GDP), which is followed by dissociation of the regulatory G*α* subunit from the catalytic G*βγ* subunit dimer. Upon activation, the G protein subunits can activate different enzymes leading to the production of inositol phosphates, protein kinase activation, an increase in intracellular Ca^2+^ production, and actin reorganization. Activation of the CXCR3 by CXCL10 leads to different cellular actions, such as chemotaxis, phagocytosis, cell degranulation, and respiratory burst [10, 43, 44]. Signaling via CXCR3 after CXCL10 binding is dependent on the type of target cell and the type of CXCR3 isoform bound to the surface of this cell.

The biological effects of CXCR3 signaling after CXCL10 between mice and humans are critically different. This is the result of differences in isoform expression in mice and humans. After the identification of CXCR3 expression in mice [45], no other isoforms are identified. In humans, the known isoforms identified are CXCR3-A, CXCR3-B, and CXCR3-alt. CXCR3-A consists of 368 amino acids and is associated with a G*α*i or a G*α*q subunit. It is widely expressed by different cell types. This isoform is similar to the CXCR3 in mice, regarding cell expression and signaling effects [46]. CXCR3-B is a larger receptor of 415 amino acids with a larger N-terminus, compared to CXCR3-A. This isoform is mainly expressed by (microvascular) endothelial cells [44]. The third isoform, CXCR3-alt, is generated by posttranscriptional exon skipping resulting in only four to five trans-membrane domains and shows a drastically altered C-terminal protein sequence. Its functions are relatively unknown, except that it is coexpressed with CXCR3-A at very low levels and that CXCL10 does not bind to this isoform and only mediates functions of CXCL11 [47].

As mentioned, CXCL10 can also exert its functions via CXCR3-independent pathways, mostly studied in an* in vitro* setting. CXCL10 is able to bind to GAGs [6] and is involved in inhibiting endothelial cell proliferation, independent of CXCR3 signaling [13]. The angiostatic properties of CXCL10, however, seem to be dependent on CXCR3 binding and not binding by GAGs [48]. Fibroblast recruitment by CXCL10 has also been linked to binding to GAGs instead of CXCR3, in which CXCL10 functions as an antifibrotic chemokine [12]. Interestingly, evidence has been found for CXCL10 signaling independent of binding to CXCR3 or GAGs, which might be related to epithelial and endothelial cell functions. The exact mechanism is not described yet [14].

### 2.2. CXCL10 Effects

CXCL10 can function via autocrine or paracrine effects. CXCL10 has versatile biological functions on different cell types, which are mostly dependent on the expression of CXCR3. Actions include attraction of inflammatory cells, such as monocytes and T lymphocytes, but CXCL10 can also function in proliferation and migration of endothelial cells and VSMCs [15–18, 49].

#### 2.2.1. CXCL10 Effects in Mice

In mice, one isoform of CXCR3 receptor is identified as mediating angiostatic effects of CXCL10, similar to the CXCR3-A receptor identified in humans [46]. Effects of CXCL10 in mice have been described extensively. These are Th1 lymphocyte recruitment, activation and attraction of B lymphocytes, macrophages, and natural killer (NK) cells to the site of inflammation [43]. Effects of CXCL10 on endothelial cells and VSMCs have rarely been described in mice. In rats, however, Wang et al. described upregulation of CXCL10 by VSMCs and reported both proliferative and chemotactic effects of CXCL10 on VSMCs [15].

#### 2.2.2. CXCL10 Effects in Humans

In humans, the effects of CXCL10 differ from those in mice. This is largely due to discrepancies in CXCR3 isoform expression between human and mice. The variety of effects of CXCL10 primarily depends on binding to CXCR3 and is therefore cell dependent. In humans, three isoforms of this receptor have been identified to which CXCL10 binds: CXCR3-A and CXCR3-B, differentially expressed by various cell types.

The first discovered isoform, CXCR3-A, is formed after the splicing of a single intron. CXCR3-A is known as the “angiostatic” isoform and is expressed by leukocytes [46, 50] and (vascular) SMCs [51, 52] and epithelial cells [53]. Functions of CXCR3-A are comparable to the CXCR3 functions in mice. Expression of the CXCR3-B or CXCR3-alt isoform has not been described in mice, suggesting that the “antiangiogenic” properties of CXCR3 only emerge in humans [18]. After binding to CXCR3-A, CXCL10 can promote cell proliferation and functions as a chemoattractant for leukocytes, especially Th1 lymphocytes [43]. CXCL10 has also recently been described in VSMC dedifferentiation during spiral artery remodeling in human VSMC cell line [54]. CXCR3-B, the “antiangiogenic” isoform, is primarily expressed by epithelial and endothelial cells. Binding of CXCL10 to CXCR3-B inhibits cell migration and promotes cell apoptosis. These data emerge from* in vitro* experiments performed with different human vascular endothelial cells, such as human umbilical cord endothelial cells (HUVECs) or human microvascular endothelial cells (HMECs) [46, 55–57].

**Figure 1:**
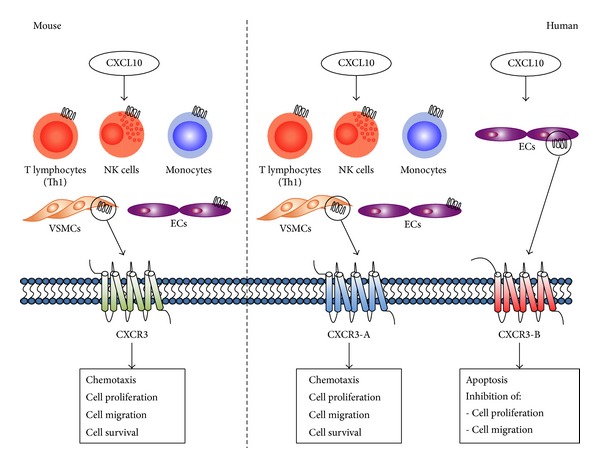
The effect of CXCL10 on CXCR3 isoforms in mouse and human tissues. Schematic overview of CXCR3 isoform expression in mouse and human cells and its actions after CXCL10 binding. In mice, one isoform of CXCR3 receptor has been identified and known as an angiostatic receptor. CXCR3 is expressed by T lymphocytes (Th1), monocytes, NK cells, VSMCs and endothelial cells (low expression levels). After binding of CXCL10, the murine CXCR3 receptor mediates cell functions, such as chemotaxis, cell proliferation, migration, and survival. In humans, this isoform is known as CXCR3-A with similar expression patterns and functions. In addition, a second isoform, known as CXCR3-B, is identified in human binding CXCL10. This isoform is primarily expressed by endothelial cells and is known for its antiangiogenic properties. These include promoting cell apoptosis and inhibiting cell proliferation and migration. CXCL10: chemokine (c-x-c motif) ligand 10; CXCR3: chemokine (c-x-c motif) receptor 3; ECs: endothelial cells; NK cell: natural killer cell; VSMCs: vascular smooth muscle cells.

The schematic Figure 1 summarizes the effects of CXCL10 on different cell types after binding to the different CXCR3 isoforms in both mouse and human.

## 3. CXCL10 in Cardiovascular Disease

### 3.1. Chemokines in Cardiovascular Disease

Vascular remodeling can be a consequence of (cardio)vascular disease and describes structural adaptation (enlargement or contraction) of the vascular lumen and vascular wall in response to various stimuli. These can be changes in blood flow leading to shear stress and hypoxia or immunological or mechanical changes leading to vascular wall damage [1, 58]. During vascular remodeling, structural changes occur in all layers of the vascular wall. These changes can result from disturbance of the structural and functional integrity of the endothelium, accumulation of inflammatory cells, and changes in SMC composition, all contributing to the severity of the disease [50, 51].

Cellular behavior in vascular tissues is directed by chemokines. Different types of chemokines and their receptors have been described to be involved in vascular remodeling. CXCL10 is known to contribute to the pathophysiology of cardiovascular disease, such as atherosclerosis, aneurysm formation, MI, and PAD, in both experimental and clinical studies.

### 3.2. CXCL10 in Atherosclerosis

Atherosclerosis is a progressive inflammatory disease occurring in the middle-sized and larger arteries that can result in gradual luminal narrowing or acute thrombotic occlusion as a result of atherosclerotic plaque rupture [59]. Initially, activated endothelial cells express adhesion molecules resulting in adhesion and infiltration of inflammatory cells, such as monocytes and T lymphocytes. During plaque progression, inflammatory cells infiltrate the vessel wall and VSMCs start to proliferate and migrate. Chemokines in general have been described to be crucial during all phases of atherosclerotic disease progression [60, 61]. The role of CXCL10 in atherosclerosis has been studied in the past. Mach et al. were of the first to describe expression of CXCL10 in human atherosclerotic plaques in different stages of the disease. Endothelial cells, VSMCs, and macrophages express CXCL10 at all examined stages of lesion development. Expression of CXCR3 could also be observed in these stages, and the vast majority of CXCR3 expressing cells was CD4^+^ T lymphocytes [62]. Shortly after this observation, mouse models for atherosclerosis revealed a role for CXCL10 in (early) lesion development. Compared to ApoE^−/−^ mice, double knockout mice for ApoE and CXCL10 demonstrated significant reductions in atherogenesis, resulting in smaller lesions. Furthermore, less CD4+ T lymphocytes accumulated in the lesions, with a simultaneous increase of regulatory T lymphocytes (T_regs_) [18]. ApoE^−/−^CXCR3^−/−^ mice also displayed significantly reduced atherosclerotic plaque development compared to ApoE^−/−^ mice, accompanied with increased numbers of T_regs_ [21]. As a result, interventions targeting plaque progression have been tested to elucidate the effect of CXCL10 inhibition* in vivo*. Treatment with a specific antagonist for CXCR3 (NBI-74330) in ApoE^−/−^ mice resulted in similar effects as in CXCR3^−/−^ mice [22]. Also, treatment of LDLR^−/−^ mice with an inhibitor for CCR5 and CXCR3 reduced atherosclerotic plaque area, T lymphocyte number, and IFN*γ* plaque expression [23]. ApoE^−/−^ mice subjected to shear stress followed by treatment with an antibody against CXCL10 resulted in a more stable plaque phenotype with twice as many SMCs compared to untreated controls but did not change overall plaque size [19]. Cheng et al. already observed the relation between shear stress, CXCL10 production, and plaque stability. Low shear stress resulted in a 10-fold higher CXCL10 mRNA expression in the vessel wall. Expression of CXCL10 was confirmed by immunohistochemical analysis, revealing that CXCL10 was mainly localized in the medial regions of the murine plaques [20]. Lastly, CXCL10 is significantly involved in VSMC proliferation and intimal hyperplasia, both important in arterial restenosis [63, 64].

### 3.3. CXCL10 in Aneurysm Formation

Aneurysm formation often coexists with advanced atherosclerotic disease [65] and systemic atherosclerosis is considered a risk factor for aneurysm formation [66]. Similar to atherosclerosis, aneurysmal tissue is characterized by inflammatory cell infiltrates, in particular B and T lymphocytes (mostly Th1) [67], which is related to aneurysm development and local thrombus formation [68]. Both inflammation and intraluminal thrombus formation have been related to aortic wall disruption and can therefore contribute to abdominal aortic aneurysm (AAA) growth and rupture [69]. Inflammatory cytokines and chemokines have been considered to play a causal role in aneurysm formation. CXCL10 and its receptor CXCR3 have been studied in mouse models for AAA, with conflicting results. King et al. observed that ApoE^−/−^CXCL10^−/−^ mice after angiotensin II infusion had worse aneurysmal disease accompanied with more dilation and rupture, suggesting a protective role for CXCL10 in AAA formation [24]. In contrast, CXCR3 signaling itself seems to be crucial for aneurysm development in wildtype mice [26]. However, these results emerge from aneurysm formation in a different vessel type (aorta versus carotid artery). Contradictory, in a different murine model for aortic aneurysm formation, CXCR3 does not seem to play a crucial role [25]. In clinical studies, CXCL10 and CXCR3 expression have been related to recruitment of CXCR3+ T lymphocytes secreting IFN*γ* and CXCL10. Furthermore, a correlation could be observed with outward arterial remodeling and intimal expansion. This remodeling resulted in matrix degradation [33]. Furthermore, differences were observed in inflammation between types of aneurysms, since CXCL10 is expressed 20-fold higher in AAA compared to popliteal artery aneurysms (PAA) [34]. Lastly, patients suffering from thoracic aortic aneurysms showed significantly elevated circulating CXCL10 levels compared to controls [26]. Although preclinical data show conflicting results, clinical studies point out a more clear direction of CXCL10 in aneurysm formation.

### 3.4. CXCL10 in Myocardial Infarction

During myocardial infarction (MI), upregulation of chemokines is a prominent feature during the postinfarction period. Several CXC chemokines are consistently upregulated in different MI models, where they play a crucial role in leukocyte trafficking and postinfarct wound healing [27]. The exact mechanisms of chemokine expression regulation are still unclear. In addition, upregulation of different chemokines at different time points during and after MI has not been fully elucidated [70]. Experimental studies have shown that CXCL10 not only functions as an angiostatic protein, but also acts asantifibrotic [5, 6, 71]. In particular, the latter finding may be crucial in postinfarct tissue healing and the accompanied fibrosis. In canine and murine MI models, CXCL10 was upregulated in the ischemic myocardial tissue during the first 24 hours [27, 28]. CXCL10 can therefore act as an angiostatic and antifibrotic chemokine to prevent premature neovascularization and fibrosis until the damaged myocardium is cleared from apoptotic and necrotic cells. Studying cardiac injury, cardiac repair, and postinfarct remodeling in CXCL10^−/−^ mice after MI suggested an essential role for CXCL10 in the infarct healing. CXCL10^−/−^ mice showed more intense inflammatory cell infiltration, cardiac remodeling, and expansion of the formed scar. The fibrotic response was also attenuated and premature compared to wildtype mice. Surprisingly, CXCL10^−/−^ mice did not show early neovascularization [29]. CXCL10 has previously been studied as a circulating biomarker and predictor of cardiovascular damage in patients suffering from (acute) MI. The use of CXCL10 as a biomarker in cardiovascular disease will be discussed further in this review.

### 3.5. CXCL10 in Collateral Artery Formation

Patients suffering from PAD often have a reduced arterial flow to the lower limbs due to local atherosclerotic plaques in the middle-sized and larger arteries. Adaptive collateral artery growth, known as arteriogenesis, is often hampered in these patients [72, 73]. The underlying mechanism of arteriogenesis is not yet fully understood, but important processes involved are local infiltration and extravasation of inflammatory cells and proliferation and migration of VSMCs [74, 75]. Chemokines are key players in arteriogenesis and have already been extensively studied (for review, see Shireman [76]). Experimental models studying arteriogenesis already proposed different chemokines involved, such as monocyte chemoattractant protein (MCP)-1, also known as CCL2 [30, 77, 78]. Earlier studies already showed the role of CXCR3 and IFN*γ* in hindlimb ischemia models. CXCR3^−/−^ mice undergoing unilateral femoral artery ligation resulted in lower perfusion recovery, accompanied with lower capillary density in the ischemic calf muscle and less infiltration of macrophages and T lymphocytes in the perivascular space in the ischemic hindlimb muscles [31]. Lee et al. showed that CXCL10 is upregulated in the hindlimb muscle tissue in the late phase of hindlimb ischemia, again suggesting the involvement of CXCL10 in neovascularization [30]. Recently, we confirmed the involvement of CXCL10 in arteriogenesis in a murine hindlimb ischemia model. Compared to wildtype, CXCL10^−/−^ mice showed significantly reduced perfusion recovery after unilateral femoral artery ligation. This was explained by significantly less collateral vessel formation and hampered enlargement of collateral vasculature. In addition, we showed that in particular CXCL10 is involved in migration of VSMCs* in vitro* [32].

### 3.6. CXCL10 as a Biomarker for Cardiovascular Disease

As described above, CXCL10 is involved in different levels of cardiovascular disease severity. In the past, clinical studies have shown that circulating chemokine levels can function as independent predictors (“biomarkers”) of (acute) ischemic cardiovascular events and cardiovascular death [79–82]. It is important to mention that a biomarker is only reliable for its function if it fulfills certain criteria, such as stability between individuals, long half-life, and easy and fast measurement using low-cost methods, and is not dependent of collection methods (e.g., serum/plasma) [83].

The function of CXCL10 as a biomarker for cardiovascular events has been investigated by measuring the transient levels of circulating CXCL10 protein during (acute) MI and treatment with percutaneous coronary intervention (PCI). In patients suffering from acute MI (AMI) CXCL10 serum levels correlated negatively to creatine kinase (CK)-MB release, a marker for MI. CXCL10 serum levels before PCI were considered as an independent predictor of CK-MB release. Furthermore, CXCL10 serum levels also negatively correlated to infarct size. Compared to healthy controls, AMI patients had significantly higher CXCL10 serum levels [40]. In contrast, in a study from Ørn et al. CXCL10 serum levels measured at the onset of an AMI correlated positively to infarct size, which did not confirm earlier data [41]. However, these studies used different methods to determine myocardial damage (CK-MB release versus Troponin I release). On the other hand, Herder et al. investigated CXCL10 serum levels along with other chemokines in a case-cohort study with patients diagnosed with coronary heart disease (CHD). The cohort consisted of 381 cases versus 1977 controls included over an 11-year time period. Although baseline CXCL10 serum levels were significantly higher in cases versus controls, adjustment for sex, age, and cardiovascular risk factors resulted in a nonsignificant contribution of CXCL10 serum measurements for risk assessment of CHD [35]. Furthermore, Ardigo et al. [37] identified CXCL10 as a potential biomarker in a much smaller cohort using a multibiomarker approach in patients suffering from CAD participating in the ADVANCE study. They report significantly higher serum CXCL10 levels in patients compared to controls (48 patients versus 44 controls). Nevertheless, measuring multiple chemokines is recommended for a more relevant biological analysis of the disease, instead of a single measurement approach of, for instance, CXCL10.

Simultaneously, Rothenbacher et al. reported a positive association between CXCL10 serum levels and risk for CHD in a clinical case-control study of almost 800 patients, even after adjustment for conventional CHD risk factors. Unfortunately, no (long-term) clinical followup was performed in these individuals [36]. Clinical studies provided important evidence for the role of CXCL10 in patients suffering from coronary artery disease (CAD). Patients suffering from unstable angina showed an increased CXCL10 expression in PBMCs as early as 6 hours after onset of complaints compared to controls or patients suffering from stable angina [38]. Furthermore, plasma CXCL10 andIFN*γ* levels have been associated with the formation of collaterals in CAD patients [39].

Patients suffering from critical limb ischemia also showed higher serum CXCL10 levels, next to increased levels of other inflammatory markers. In addition, they showed a negative correlation between inflammatory cytokines and CD34+ bone marrow cells. Teraa et al. discussed that prolonged exposure to proinflammatory stimuli may lead to exhaustion or suppression of the CD34+ cell pool in the bone marrow [42]. It is important to mention that the measured circulating CXCL10 levels in different studies mostly showed a large variation, which is an important indicator of a low reliability of this approach. Because single CXCL10 measurements did not result in reliable predictions, the described studies all recommended a combined chemokine approach for cardiovascular risk prediction. However, this approach seems unrealistic from a practical and clinical point of view for obvious reasons, such as variability and costs. Although chemokines have been extensively described as pathogenic key players in cardiovascular disease, this does not automatically mean that they are suited as biomarkers or risk predictors. Furthermore, the use of chemokines as biomarkers in cardiovascular risk prediction brings some challenges. As mentioned, a biomarker needs to fulfill criteria regarding its reliability. Chemokines as biomarkers are challenged by their stability, half-life, level fluctuation with use of different anticoagulants, and their low concentrations [83]. CXCL10 has been described to bind locally to endothelial cells or extracellular matrix components via GAGs, which makes the function of CXCL10 as a reliable circulating biomarker questionable.

In the past, genetic variations in chemokine genes have been extensively described, for example, for MCP-1, CCL2, and 5 (for reviews, see [83, 84]). The mechanism by which the 9p21 locus is associated with CAD and other cardiovascular diseases has been investigated in the past by, for instance, the effect on impaired IFN*γ* responses [85]. However, this mechanism does not involve the regulation of CXCL10 responses itself [86]. Until now, no genetic variations in the CXCL10 gene have been linked to cardiovascular risk prediction.

Table 1 provides an overview of the role of CXCL10 in cardiovascular disease in experimental and clinical settings.

## 4. Summary and Conclusion

In this review, we summarized the role of CXCL10 in different cardiovascular diseases, in both the experimental and clinical setting. The role of CXCL10 is rather complex, also depending on its action on different isoforms of the CXCR3 receptor. Since CXCR3 is differentially expressed by different cell types, which also differs between mouse and man, translating experimental and clinical data is challenging. Also other signaling pathways, independent of CXCR3, have been described. The involvement of CXCL10 in atherosclerosis in both experimental and clinical settings suggests that CXCL10 contributes positively to the initiation and progression of the disease. In contrast, the functions of CXCL10 in aneurysm formation and MI are less consistent. This can be partly explained by study design and possible (biological) discrepancies between animal models and patient groups studied. Animal models for PAD suggest that CXCL10 is positively involved in arteriogenesis. Both CXCR3 and CXCL10 deficiencies resulted in decreased perfusion recovery in a murine hindlimb ischemia model. To this date, clinical evidence for the role of CXCL10 in PAD patients is scarce.

CXCL10 as a biomarker for cardiovascular risk prediction has been investigated in different clinical studies. However, measuring a single marker to predict cardiovascular risk will not be conclusive, which holds true for most potential biomarkers. Furthermore, CXCL10 as a reliable biomarker, regarding its stability, consistency, and possible binding to GAGs, is suboptimal. Lastly, CXCL10 is significantly involved in VSMC proliferation and intimal hyperplasia, both important in arterial restenosis. In particular, since other inflammatory diseases are also linked to CXCL10, further research is needed to elucidate the effects of CXCL10.

## Figures and Tables

**Table 1:** The role of CXCL10 (and CXCR3) in cardiovascular disease—experimental and clinical studies.

Clinical setting	Animal model	Experimental outcome	References
Atherosclerosis	ApoE^−/−^CXCL10^−/−^ mice	CXCL10^−/−^ mice had smaller a plaque size compared to ApoE^−/−^	Heller et al. [18]
	ApoE^−/−^ mice, CXCL10 antibody treatment	CXCL10 inhibition attenuated vulnerable plaque formation without size reduction compared to placebo	Segers et al. [19]
	ApoE^−/−^ mice, carotid artery cast model	Low shear stress increased CXCL10 mRNA tissue expression compared to normal shear stress	Cheng et al. [20]
	ApoE^−/−^CXCR3^−/−^ mice	CXCR3^−/−^ mice had more stable plaques and smaller plaques compared to ApoE^−/−^ mice	Veillard et al. [21]
	ApoE^−/−^ mice, CXCR3 antagonist treatment	CXCR3 antagonist treatment attenuated vulnerable plaque formation and growth compared to placebo	Van Wanrooij et al. [22]
	LDLR^−/−^CXCR3^−/−^ mice	CXCR3^−/−^ mice had a reduced plaque size compared to LDLR^−/−^ mice	Van Wanrooij et al. [23]

Aneurysm formation	ApoE^−/−^CXCL10^−/−^, angiotensin II infusion	CXCL10^−/−^ mice had larger and more rupture-prone aneurysms compared to ApoE^−/−^ mice	King et al. [24]
	CXCR3^−/−^ mice, Calcium Chloride infusion	CXCR3^−/−^ mice had no reduction in aneurysm formation compared to wildtype mice	MacTaggart et al. [25]
	CXCR3^−/−^ mice, Calcium Chloride infusion	CXCR3^−/−^ mice had reduced aneurysm formation compared to wildtype mice	Gallo et al. [26]

Myocardial infarction	Dogs, myocardial I/R	Upregulated CXCL10 mRNA expression 24–48 h after reperfusion in myocardial tissue	Frangogiannis et al. [27] Dewald et al. [28]
	Wildtype mice, myocardial I/R	Upregulated CXCL10 mRNA expression 6 h after reperfusion in myocardial tissue	Dewald et al. [28]
	CXCL10^−/−^ mice, myocardial I/R	CXCL10^−/−^ mice had a reduced inflammatory, angiogenic, and fibrotic response compared to wildtype	Bujak et al. [29]

Collateral artery formation	Wildtype mice, hindlimb ischemia	Upregulated CXCL10 mRNA expression in ischemic muscle tissue after hindlimb ischemia	Lee et al. [30], Waeckel et al. [31]
	CXCL10^−/−^ mice, hindlimb ischemia	CXCL10^−/−^ mice had a reduced perfusion recovery compared to wildtype mice	Van den Borne et al. [32]
	CXCR3^−/−^ mice, hindlimb ischemia	CXCR3^−/−^ mice had a reduced perfusion recovery compared to wildtype mice	Waeckel et al. [31]

Clinical setting	Patient population	Clinical outcome	References

Aneurysms	Thoracic aortic aneurysms	CXCL10 expression is correlated to outward remodeling and matrix degeneration	Tang et al. [33]
	Abdominal aortic versus popliteal artery aneurysm	Significant differences in CXCL10 expression levels between aneurysm subtypes	Abdul-Hussien et al. [34]
	Thoracic aortic aneurysms (case-control study)	Higher circulating CXCL10 levels in cases versus controls	Gallo et al. [26]

Coronary artery disease (CAD)	CAD	Higher baseline CXCL10 levels in cases versus controls, not predictive for CV risk	Herder et al. [35]
	CAD	Higher baseline CXCL10 levels in cases versus controls, no followup performed	Rothenbacher et al. [36]
	CAD	Higher baseline CXCL10 levels in cases versus controls, not predictive for CV risk	Ardigo et al. [37]
	Unstable angina	Higher CXCL10 expression in cases versus controls 6 h after complaints	Oliveira et al. [38]
	Collateral formation	Increased collateral formation was associated with higher CXCL10 plasma levels	Keeley et al. [39]

Myocardial infarction	Acute MI	Negative correlation between serum CXCL10 and markers for MI and infarct size	Koten et al. [40]
	Acute MI	Higher serum CXCL10 levels in acute MI patients versus healthy controls	Koten et al. [40]
	Acute MI	Positive correlation between serum CXCL10 levels and infarct size	Ørn et al. [41]

Peripheral artery disease (PAD)	Critical limb ischemia	Higher CXCL10 serum levels in patients versus controls	Teraa et al. [42]
